# Neuroimmune mechanisms in autism etiology - untangling a complex problem using human cellular models

**DOI:** 10.1093/oons/kvae003

**Published:** 2024-02-22

**Authors:** Janay M Vacharasin, Joseph A Ward, Mikayla M McCord, Kaitlin Cox, Jaime Imitola, Sofia B Lizarraga

**Affiliations:** Department of Biological Sciences, and Center for Childhood Neurotherapeutics, Univ. of South Carolina, 715 Sumter Street, Columbia, SC 29208, USA; Department of Biological Sciences, Francis Marion University, 4822 East Palmetto Street, Florence, S.C. 29506, USA; Department of Molecular Biology, Cell Biology, & Biochemistry, Brown University, 185 Meeting Street, Providence, RI 02912, USA; Center for Translational Neuroscience, Carney Institute of Brain Science, Brown University, 70 Ship Street, Providence, RI 02903, USA; Department of Biological Sciences, and Center for Childhood Neurotherapeutics, Univ. of South Carolina, 715 Sumter Street, Columbia, SC 29208, USA; Department of Biological Sciences, and Center for Childhood Neurotherapeutics, Univ. of South Carolina, 715 Sumter Street, Columbia, SC 29208, USA; Laboratory of Neural Stem Cells and Functional Neurogenetics, UConn Health, Departments of Neuroscience, Neurology, Genetics and Genome Sciences, UConn Health, 263 Farmington Avenue, Farmington, CT 06030-5357, USA; Department of Molecular Biology, Cell Biology, & Biochemistry, Brown University, 185 Meeting Street, Providence, RI 02912, USA; Center for Translational Neuroscience, Carney Institute of Brain Science, Brown University, 70 Ship Street, Providence, RI 02903, USA

**Keywords:** MIA, ASD, iPSCs, Epigenetics, Pro-inflammatory cytokines, Organoids, Neuroinflammation

## Abstract

Autism spectrum disorder (ASD) affects 1 in 36 people and is more often diagnosed in males than in females. Core features of ASD are impaired social interactions, repetitive behaviors and deficits in verbal communication. ASD is a highly heterogeneous and heritable disorder, yet its underlying genetic causes account only for up to 80% of the cases. Hence, a subset of ASD cases could be influenced by environmental risk factors. Maternal immune activation (MIA) is a response to inflammation during pregnancy, which can lead to increased inflammatory signals to the fetus. Inflammatory signals can cross the placenta and blood brain barriers affecting fetal brain development. Epidemiological and animal studies suggest that MIA could contribute to ASD etiology. However, human mechanistic studies have been hindered by a lack of experimental systems that could replicate the impact of MIA during fetal development. Therefore, mechanisms altered by inflammation during human pre-natal brain development, and that could underlie ASD pathogenesis have been largely understudied. The advent of human cellular models with induced pluripotent stem cell (iPSC) and organoid technology is closing this gap in knowledge by providing both access to molecular manipulations and culturing capability of tissue that would be otherwise inaccessible. We present an overview of multiple levels of evidence from clinical, epidemiological, and cellular studies that provide a potential link between higher ASD risk and inflammation. More importantly, we discuss how stem cell-derived models may constitute an ideal experimental system to mechanistically interrogate the effect of inflammation during the early stages of brain development.

## Teaser

ASD affects 1 in 36 people and its core features are impaired sociability, the presence of repetitive behaviors and deficits in verbal communication. While most ASD cases are of genetic origin there is a large number of cases that are of complex or unknown etiology, which suggests the potential for environmental risk factors to influence ASD pathogenesis. In fact, a large number of studies that include epidemiology and animal studies suggest that severe infections during pregnancy could lead to a higher risk of ASD in the progeny. However, studies in human experimental systems that allow for the dissection of precise mechanisms during early brain development which could be affected due to inflammation have been lacking. Stem cell technology gives us the opportunity to study the early stages of human brain development. Here we broadly discuss clinical, epidemiological, and mechanistic studies that focus on the effect of inflammation in the developing brain, and we review recent work using iPSC-based models that study how pre-natal inflammation during the early stages of brain development could increase ASD risk.

## INTRODUCTION

Autism spectrum disorders (ASD) are a highly heterogeneous and heritable class of disorders with complex or unknown genetic etiology [1]. Environmental risk factors could contribute to the complex or unknown etiology of ASD in a subset of cases. Evidence from large epidemiological studies suggest that excessive immune activation due to maternal infection during pregnancy could be a major environmental risk factor for ASD [2, 3]. For instance, in humans, severe viral infection during pregnancy has been correlated with a 30% increase in ASD risk in the progeny [3]. However, the mechanisms underlying the increased risk in ASD associated with inflammation are unclear and suggest a para-infectious mechanism rather than a direct viral infection of the fetus. Similarly, clinical studies suggest a higher correlation between autistic regression and predisposition to autoimmunity [4]. Further evidence supporting the connection between immune alterations and ASD pathogenesis is provided by postmortem brain studies that suggest altered expression of microglial genes and innate immune response genes in brains from ASD patients [5].

**Figure 1:**
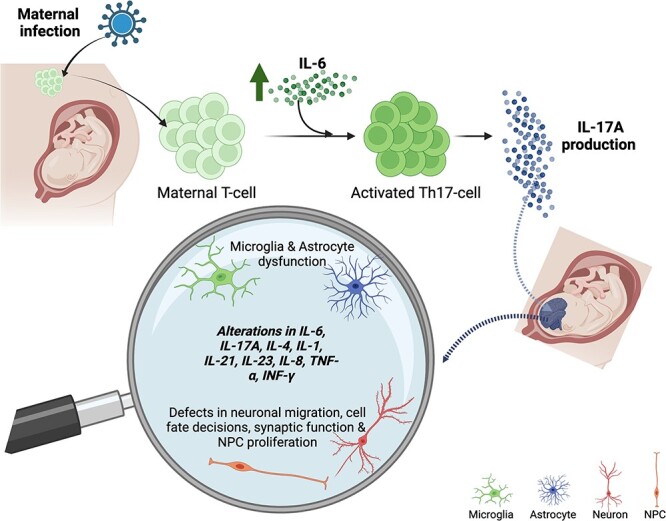
**Maternal immune activation mechanisms that could underlie ASD pathogenesis.** A representation of how maternal inflammation during pregnancy can lead to alterations in the fetal brain is shown. During pregnancy, exposure to an inflammatory agent like a virus (globular shape with spikes) could lead to the release of pro-inflammatory cytokines like IL-6. Elevated levels of IL-6 will lead to the activation of Th17 cells that produce the pro-inflammatory cytokine IL-17A. IL-17A can cross the placental barrier affecting fetal brain development. IL-6 receptors present in microglia suggest that they can also be directly activated by IL-6. Effects of elevated cytokine levels during fetal brain development could include increased levels of other cytokines leading to the activation of microglia and astrocytes, which could in turn alter the function and phenotypes of NPCs and/or neurons

**Table 1:** Epidemiological studies of MIA and ASD risk association discuss in this review

Cohort information	Study Design	Results	Citations
1' 206, 600 children followed up to 20 years of age in Denmark	Retrospective study from 1996–2015. Information gathered through the Danish Civil Registration System; infection/antibiotic information gathered through hospital contacts (National Patient Register)	↑ psychological and mental disorders association w/exposure to infections & antibiotics peri- and post-natal	[2]
2' 371, 403 persons with 24 414 ASD cases,	Retrospective study from Swedish nationwide birth cohort registry born 1984–2007. A total of 903 mothers had an in-patient diagnosis of infection during pregnancy.	30% ↑ ASD risk associated with inpatient diagnosis of infection	[3]
96, 736 children between 8–14 years old in Denmark	Retrospective study from 1997–2003. Infection information gathered through phone interviews. ASD diagnoses gathered through Danish Psychiatric Register. Severity of infection was determined by length of illness and types of treatment received	2x ↑ ASD risk associated with severe infections.	[35]
243 children in US with congenital rubella in 1967.	Longitudinal study. Children with rubella were examined and diagnosed based on behavioral descriptions and reports from home, school, and in-person observations	↑ ASD risk.	[36]
70 children with proven congenitally-infection with CMV born between 2007–2012.	Retrospective study with prospective data collection correlated CMV infection with ASD prevalence.	2–3-fold ↑ prevalence of ASD in CMV infected children compared to general population	[38]
69 children under 18 years old with CMV and 292 children with non-CMV infections between 2006–2012 in China	Retrospective study examined records for onset for mental disorders later in life for children that had CMV testing within 3 weeks of age.	↑ risk of ASD and epilepsy in children with CMV infection compared to children with non-CMV infections	[39]
235 children born in Guadeloupe, Martinique, or French Guiana from mothers whose pregnancies overlapped with 2016 ZIKV epidemic.	Population-based mother–child cohort study. Normocephalic newborns exposed in utero (156) or not exposed in utero (156) to ZIKV. Neurodevelopment assessment conducted (ASQ) at 2 years of age between 2018–2019.	↓ communication skills, ↓ social skills but low rate of ASD diagnosis because of age	[42]
216 children born from women infected with ZIKV during pregnancy from 2015–2016 in Rio de Janeiro, Brazil.	Retrospective study. Neurodevelopmental Bayley-III (bayle scales of infant or todler development ED-3) or HINE examinations (Hammersmith infant neurological examinations) conducted on 146 children at a median of 18 months of age and through neurodevelopmental questionnaires/neurological examinations on 70 children	↓ cognitive ability ↓ language function (most affected) ↑ birth defects such as eye abnormalities and hearing deficits	[43]
7,772 children born between March – September 2020 in Massachusetts.	Retrospective study including 7550 control unaffected mothers and 222 SARS-CoV-2 infected mothers. Neurodevelopmental deficiencies analyzed from ICD-10 (international classification of diseases tenth revision) during the first year of life	↓ motor function ↓ speech ↓ language skills ↑ risk of neurodevelopmental disorders in first year of birth	[47]
18, 355 children from 17, 478 live births of unaffected mothers and 877 SARS-CoV-2 positive mothers in Massachusetts.	Retrospective study included live offspring of mothers who delivered in 2018 and followed before COVID-19 pandemic, in 2019 followed up during the COVID-19 pandemic, and in 2020 to 2021 followed up during the COVID-19 pandemic. Children were followed up with 1 year after birth and examined for neurological deficiencies	↓ motor function in males ↓ communication skills in males	[48]

Representative studies are shown for general infection, rubella, cytomegalovirus (CMV) infections, ZIKA virus (ZIKV) infections and SARS-COV-2 infections.

Animal models of Maternal immune activation (MIA) provide construct validity to the study of ASD in terms of behavioral phenotypes as well as the study of sex-specific differences associated with ASD [6–12]. Studies on the effect of influenza infection during pregnancy in rhesus monkeys showed a significant reduction in cortical gray matter [13]. In healthy adults, intelligence has been associated with larger gray matter volume [14]. Therefore, the reduction in cortical gray matter identified in rhesus monkeys exposed to influenza [13] could parallel human cases of profound ASD that are co-morbid with intellectual disability [15]. Three core features of ASD in humans are deficits in socialization, hyper anxiety in novel or stressful situations and hypersensitivity to stimuli. Analogously, the offspring of mice infected with influenza during pregnancy showed altered brain histology that correlated with decreased sociability, increased anxiety-like behaviors, and pre-pulse inhibition deficits associated with the acoustic startle response [16]. Animal phenotypes induced by MIA further resemble previously reported human ASD phenotypes as they both share increased startle response [16, 17]. Similarly, the male bias in ASD is recapitulated in MIA male offspring as they showed reduced sociability and increased repetitive behaviors, as well as increased anxiety-like behaviors compared to female offspring [11, 18–20]. Therefore, offspring exposed to MIA tend to display ASD-like behaviors indistinctively of the species, suggesting that the responses to inflammation during neurodevelopment might be an evolutionarily conserved pathogenic mechanism.

Activation of the immune system in animal models of MIA has been shown to correlate with elevated levels of pro-inflammatory cytokines including interleukins IL-6, IL-17A, IL-1β, IL-10 and cytokines - Tumor necrosis Factor − 1 alpha (TNF-1α) and Interferon gamma (IFN-γ) [21]. However, studies in children with ASD provide contradicting evidence with respect to IL-10 findings in animal models, as decreased levels of IL-10 were reported in two independent clinical studies [22, 23]. Importantly, the IL-6/IL17A immunoregulatory axis appears to be a major mechanism of MIA in the developing brain. For example, animal models mimicking severe viral infection during pregnancy elicit immunological activation in the mother, resulting in elevated levels of IL-6, which leads to differentiation of Th0 cells into Th17 cells in the mother [10]. These IL-6 induced Th17 cells in turn produce higher levels of IL-17A [24], which can cross the placental barrier and may affect fetal brain development by activating microglia, the resident macrophage population in the brain (Fig. 1). In fact, MIA induces widespread transcriptional changes that lead to the differential expression of microglial and synaptic genes which could underlie the ASD-like behavioral phenotypes identified in MIA animal models [25–27]. Two common cellular phenotypes that could underlie ASD-like behaviors across MIA animal models are reduced synaptic density as well as impaired excitatory and inhibitory synaptic transmission [8, 28]. A long-standing hypothesis in the ASD field is that the imbalance between excitation and inhibition (E/I) disrupts normal neuronal circuitry and underlies ASD pathogenesis [29]. One potential outcome of MIA is a delay in the excitatory to inhibitory switch in gamma aminobutyric acid (GABA), which in turn could alter the E/I balance early in development and contribute to ASD pathogenesis [30].

Mechanistic understanding of how inflammation could alter fetal human brain development has lagged behind animal studies. Therefore, experimental systems that mimic the effect of inflammation or immune alterations during early human neuronal development are woefully needed. Human iPSCs are ideal for these types of studies, as they provide a fully human genome that can capture the genetic background of disease relevant cell types and allow cellular and molecular manipulations [31–34]. Here we review current evidence supporting a role for immune dysfunction in the etiology of ASD; and we conduct an in-depth discussion of how current stem cell-based models could bridge the mechanistic gap of knowledge with respect to how inflammation may disrupt early human brain development.

## EPIDEMIOLOGICAL STUDIES LINKING INFLAMMATION TO ASD

It is important to highlight that independent of the infectious agent it is the sustained inflammatory response that is triggered in the mother what is suspected to disrupt normal fetal brain development. Human epidemiological studies have linked maternal infection during prenatal stages to increased risk for ASD [2, 3, 35] (Table 1). A study of over 96, 000 Danish children identified a two-fold increased risk of ASD associated with severe maternal influenza infection [35]. Similarly, analysis of over 1.2 million Danish children showed increases in neurological disorders, including ASD, in response to maternal infections treated with anti-infective agents [2]. Specific infections associated with increased ASD risk include congenital rubella [36], and congenital cytomegalovirus (CMV) infections [37] (Table 1). ELISA analysis of umbilical cord blood or blood collected at birth showed association of congenital CMV infection with variable increased ASD risk (2.3 to 7.4%) [38, 39]. Within the last decade we observed the devastating effects on the nervous system by the ZIKA Virus (ZIKV) epidemic and COVID virus SARS-Covid-2 Virus (SARS-CoV-2) pandemic, but their direct connection to ASD risk is unknown. Next, we discuss neurodevelopmental outcomes for children exposed in utero to either ZIKV or SARS-CoV-2.

**Table 2:** Studies of prenatal inflammation by cytokine profiling

Cohort size	Population type	Type of study	Bio-material analyzed	Cytokines tested	Results	Citations
788 pairs	Mother–child pairs	Prospective birth cohort. Children evaluated with SCQ^a^ and SRS^b^ at 7 years of age to obtain ASD information. Association between maternal cytokines and ASD symptoms evaluated by multivariate linear regression.	Mother’s serum measured at 28 weeks’ gestation.	13, including IL-1β, IFN-γ, TNF-α, IL-10, IL-4, and IL-6	↑ levels of IL-4: ↑ ASD like behavior in children	[52]
331 ASD cases and 698 controls	ASD diagnosed cases	Study adjusted for maternal autoimmune disorders and maternal infections during pregnancy. Clinical data from nationwide registers.	Amniotic fluid samples	IL-4, IL-10, TNF-α, TNF-β	↑ IL-4, IL-10, TNF-α, TNF-β: correlation with ↑ ASD risk in children	[53]
84 pairs	ASD diagnosed cases	Retrospective included women who gave birth to child ultimate diagnosed with ASD.	Mid-gestational mother’s banked serum	17 cytokines and chemokines (including IL-4, IL-5, and IFN-γ)	↑ IL-4, IL-5, IFN-γ: ↑ in child’s ASD risk	[54]

Each study is an independent retrospective population study. Abbreviations: ^a^ Social Communication Questionnaire; ^b^ Social Responsiveness Scale

### The ZIKV epidemic and ASD risk

Retrospective studies on children exposed in utero to ZIKV suggest that there may be a correlation between ZIKV infection and increased neurodevelopmental outcomes that could confer ASD risk [40–42] (Table 1). To minimize the confounding factor of the severe microcephaly associated with prenatal ZIKV infection, the majority of the studies were done on children that, despite being exposed to ZIKV in utero, were normocephalic. Impairments in language development which is often associated with ASD was present in 30.7% of normocephalic children exposed to ZIKV in utero; however, only 0.7% were diagnosed with ASD [41]. Another longitudinal study of children exposed in utero to ZIKV showed close to 45% of the children had language processing deficits, while only 2.1% of them presented with ASD [43] (Table 1). However, this study did not include a control population of typically developing children. A study of 156 normocephalic 2-year-old participants exposed in utero to ZIKV showed impaired communication and social skills compared to unaffected toddlers [42]. While deficits in communication and social skills are core features of ASD, 2 years of age is considered early for a definitive ASD diagnosis. Therefore, larger longitudinal studies in ZIKV cohorts are needed to clearly establish the contribution of in utero ZIKV infection to increase risk in ASD [42].

### SARS-CoV-2 pandemic and ASD risk

Recent studies suggest that SARS-CoV-2 infection leads to a strong immune activation at the maternal-fetal interface that could lead to neurodevelopmental defects in the offspring [44–46] (Table 1). Analysis of electronic records from 7,772 live births showed that 3.3% of children exposed to SARS-CoV-2 in utero were more likely to have defects in motor function, speech and language compared to control cases [47]. Similarly, an analysis of records from 18 ,355 live births was conducted 12 months after birth on progeny from mothers tested for SARS-CoV-2 during pregnancy [48]. These reports suggested that male progeny of mothers exposed to SARS-CoV-2 while pregnant had a higher risk of motor and speech delays than female offspring [48]. Due to the participants age, these studies cannot conclusively diagnose ASD for children exposed in utero to SARS-CoV-2, yet the language and speech abnormalities identified suggest that studies of older children might uncover increased ASD risk in these cohorts.

## IDENTIFIYING ASD INFLAMMATORY SIGNATURES

A key question in the ASD field is whether there are shared inflammation signatures or immune profiles that could serve as predictive biomarkers for ASD or neurodevelopmental disease risk. We present an overview of studies focusing on the association of ASD with specific inflammatory profiles during pregnancy (Table 2) as well as in individuals that present with ASD [49–51] (Table 3).

**Table 3:** Population studies on ASD patient immune profile

Cohort Size	Population type	Bio-material	Cytokines tested	Results	Citations
888 children (370 ASD, 378 TD, and 140 DD)	Population based case control study of perinatal biomarkers for ASD.	Newborn Bloodspot analysis	IL-6, IL-8	↑ IL-6 and IL-8 receptors in ASD individuals compared to controls	[59]
80 children with ASD, 51 unaffected siblings, and 86 unrelated healthy controls	12 years and under	Venous blood samples	IL-6, IL-8, IL-9, IL-10, and TNF-α	↑↑ TNF-α, IL-8, and IL-6 in ASD individuals compared to controls	[56]
41 TD children, and 87 ASD children	Ages 1–6	Blood plasma	11 cytokines measured	↑TNF-α and ↑TGF-β in children with ASD	[57]
97 ASD cases, 87 confirmed TD, and 39 DD cases.	2- to 5-year-old children recruited as part of the population-based case–control CHARGE ^c^ study	Venous blood samples from children with ASD or TD ^a^, or DD ^b^ without autism	GM-CSF, IFN-γ, IL-1β, IL-2, IL-4, IL-5, IL-6, IL-8, IL-10, IL-12(p40), IL-13, and TNF-α	↑↑ IL-6, IL-8, IL-1β, and IL-12p40 in ASD individuals compared to TD group	[58]
28 ASD children and 28 matched controls	7–15 years old males	Blood plasma	48 analytes, including IL-1β, IL-4, IL-6, IL-8, IL-17, IFN-γ, TNF-β	↑ IL-1β, IL-1RA, IL-5, IL-8, IL-12, IL-13, IL-17, GRO-α in ASD individuals compared to controls	[60]
45 children with autism and 40 matched healthy controls	Cross-sectional study in Saudi Arabia. ASD group 36 male and 9 females between ages of 6–11 years	Serum measured	IL-17A levels measured	↑ IL-17A in ASD cases and levels correlated with severity of ASD.	[61]

Each group is a separate study of children diagnosed with ASD. Abbreviations: Typically developing ^a^; developmental delay ^b^; Childhood Autism Risk from Genetics and Environment ^c^

### Inflammatory profiles during pregnancy and ASD association

A prospective study of 788 mother–child pairs used the serum of mothers at 28 weeks of gestation to measure 13 inflammatory markers, that were correlated with neurobehavioral assessment in their progeny at 7 years of age [52]. This study identified a correlation between increased levels of IL-4 with increased ASD behaviors without adjusting for inflammation during pregnancy [52]. Additionally, a study of 331 ASD cases adjusted for maternal autoimmune disorders or infections during pregnancy identified elevated levels of IL-4, IL-10, TNF-α and TNF-β in the amniotic fluid of children later diagnosed with ASD [53]. Furthermore, comparison of banked serum obtained from mid-gestational mothers of 84 children diagnosed with ASD showed significant association between increased ASD risk and increased levels of IL-4, IL-5 and INF-γ [54]. In the brain, receptors for IL-4, IL-10 and TNF-α are highly expressed in microglia, the resident immune cell in the brain [55]. Therefore, elevated cytokine levels during pregnancy could affect the developing fetal brain by directly acting on the microglia.

### Immune profiles in ASD individuals

Studies of either serum, plasma or blood of ASD patients have provided additional evidence for differential inflammatory signatures associated with ASD (Table 3). Increases in TNF-α [56, 57], IL-8 [56, 58], IL-1β, and IL-6 [58], as well as cytokine receptors for IL-6 and IL-8 [59] were identified in ASD children compared to typically developing children. Similarly, the plasma of ASD individuals compared to unaffected individuals showed elevated levels of IL-17A [60, 61], with higher levels of IL-17A present in cases with more profound ASD (67.9%) [61]. It is important to point out that these studies were done in participants with no known genetic etiology, so the contribution of specific genetic lesions to the studies reviewed above is unknown. While there are alterations in cytokine levels associated with ASD, the high variability between different studies highlights the need for larger prospective studies coupled with carefully controlled clinical history of infections for children-mother pairs at the time of sample collection, as well as additional genetic studies on these populations. Taken together, this data brings forward two important ideas: (**1**) pre-natal inflammation could affect critical neurodevelopment events, and (**2**) post-natal inflammation may alter mechanisms essential for the fine-tuning of neuronal circuitry. In both scenarios, mechanisms relevant to the development and/or establishment of proper neuronal connectivity could be impaired and contribute to ASD pathogenesis.

## MOLECULAR MECHANISMS BRIDGING INFLAMMATION AND ASD

The identification of alterations in cytokine levels in ASD individuals raises two major questions: (**1**) Do transcriptomic changes in genes relevant to immune function underlie ASD pathogenesis? and (**2**) Does inflammation elicit specific molecular signatures in the developing brain? Next, we discuss transcriptomic and epigenetic mechanisms in relation to MIA and ASD pathogenesis.

### Transcriptomic profiling reveals immune signatures might contribute to ASD pathogenesis

Targeted profiling in blood samples of a cohort of 30 ASD patients and 41 control individuals showed higher gene expression in TNF-α, IL-6 and IL-17 in ASD patients [62]. Alterations on markers of inflammation at the gene expression and protein levels in blood from ASD patients do not necessarily reflect changes in brain tissue. However, cytokines can cross the blood–brain barrier (BBB), suggesting that they could directly exert their effects in neural cells. In fact, brain tissue studies of ASD patients showed *in situ* activation of microglia. Genome-wide transcriptional profiling of postmortem ASD brains and genome wide association studies (GWAS) identified an overrepresentation of genes involved in immune function in ASD [63]. Similarly, studies in rats on the immediate effect of MIA during early gestation showed upregulation of immune response genes that correlated with significant downregulation of genes important for synaptic function and brain development [64]. Transcriptional alterations in immune regulatory genes might account for only a subset of ASD cases, as large genetic studies in ASD show an overrepresentation of genes encoding chromatin and transcriptional regulators that have high risk variants associated with ASD [65–68]. However, the mechanistic link between inflammatory pathways and chromatin regulatory or epigenetic mechanisms with respect to ASD pathogenesis remains underexplored.

### Interrogating the intersection between chromatin regulatory mechanisms and transcriptional control in ASD and inflammation

**Figure 2:**
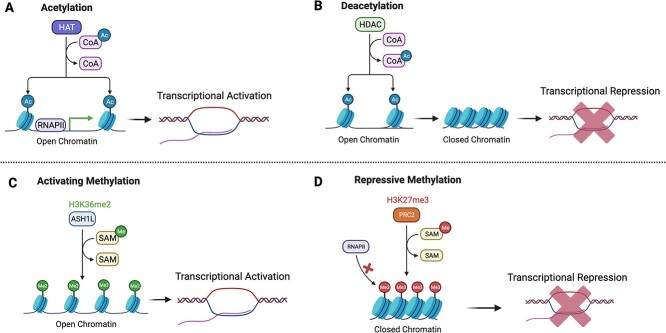
**Chromatin environment influences transcriptional control.** Representative mechanisms of chromatin regulation by post-translational modifications of histones are shown. (**A**) Histone acetylation is mediated by histone acetyltransferases that deposit an acetyl group on the histone tails. Acetylation of histones commonly leads to a chromatin environment that is permissive for transcriptional activation. (**B**) Histone de-acetylation by histone deacetyl transferases remove acetyl groups from histone tails and leads to a compacted chromatin environment that commonly leads to transcriptional repression. (**C**) Histone di-methylation on lysine 36 of histone H3 (H3K36me2) by ASH1L is shown as a representative activating mark. H3K36me2 leads to an open chromatin environment that is permissive for transcriptional activation. (**D**) Histone tri-methylation on lysine 27 of histone H3 (H3K27me3) by the Polycomb repressor complex 2 (PRC2) is shown as a representative repressive histone mark. H3K27me3 leads to a compacted chromatin environment that promotes transcriptional repression

Transcriptomic changes elicited by MIA [12, 27] could reflect epigenetic mechanisms at play. For instance, alterations in the accessibility of the chromatin could influence the transcriptional state of thousands of genes at a time with vast consequences on the developing brain. In the following sections we discuss studies that bridge ASD pathogenesis and inflammation with two broad chromatin regulatory mechanisms: DNA methylation and post-translational modification of histones (Fig. 2).

#### DNA methylation in ASD and inflammation

DNA methylation on CpG islands recruits proteins that regulate gene expression. DNA hypermethylation is associated with repression of gene expression whereas DNA hypomethylation is associated with actively transcribed genes. Studies of postmortem brain tissue from idiopathic ASD individuals and MIA animal models identified genome wide changes in DNA methylation on genes involved in synaptic signaling, immune function, neuronal regulation, as well as ASD risk genes [69, 70]. Injection of early gestating rats with polynosinic-polycytidilic acid [Poly (I:C)] – a double-stranded RNA (dsRNA) analog that mimics viral infection – induced hypomethylation at the transcriptional repressor methyl-CpG-binding protein 2 (*MeCP2*) promoter in the offspring [71]. Increased levels of MeCP2 at the promoters of genes that control the synthesis of the inhibitory neurotransmitter GABA (GAD1 and GAD2) correlated with DNA hypermethylation which lead to their transcriptional repression [71]. Similarly, there is a clear association between GABAergic dysfunction and *MECP2* mutations in syndromic ASD [72]. In contrast, analysis of postmortem frontal cortex of 14 idiopathic ASD brain samples showed DNA hypermethylation at the *MECP2* promoter, which could lead to its repression [73], and is opposite of what has been reported in MIA animals [71, 74]. However, the small sample size and unknown ASD etiology of the postmortem samples used, render these studies inconclusive with respect to the effect of MIA on DNA methylation in the developing human brain. Thus, studies that interrogate changes in the DNA methylome using human cellular models of MIA are needed to distill the impact of inflammation on DNA methylation in brain tissue.

#### Chromatin regulators in ASD and inflammation

Post-translational histone modifications provide a code to regulate gene expression by altering chromatin accessibility (Fig. 2). For example, hyper-acetylation of histones leads to an open chromatin state that correlates with transcriptional activation while hypo-acetylation of histones results in a more compacted chromatin state that promotes transcriptional repression [75]. Several histone deacetylases (HDACs) have been previously associated with neuroinflammation [76] and broad inhibition of HDACs by Valproic acid is a major environmental risk factor for ASD [77]. Therefore, dysregulation of histone acetylation could be a major molecular mechanism that underlies the effect of MIA in the developing fetal brain. In fact, animals prenatally exposed to MIA showed decreased levels of global histone acetylation in the cerebral cortex, which correlated with decreased expression of genes important for neuronal development and immune signaling [78]. Postmortem studies of ASD brain tissue identified alterations in histone acetylation across multiple regulatory regions in genes associated with ASD risk [79, 80], but to date there is no direct mechanistic link between MIA and histone acetylation in human brain tissue.

**Figure 3:**
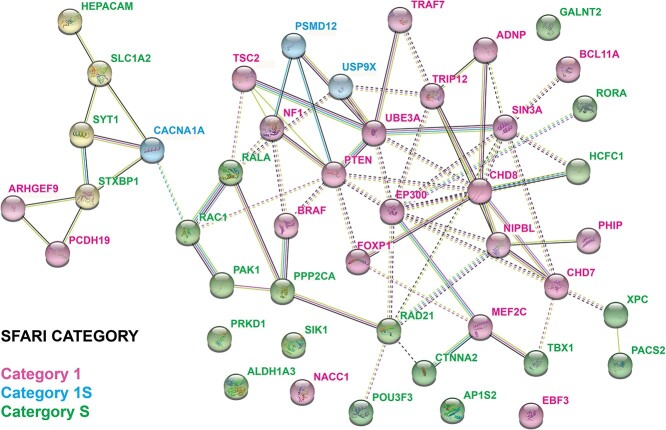
**Intersection of ASD risk genes and immune pathways.** Network representation of ASD risk genes identified after comparative analysis of the SFARI human gene and the immune portal databases. Genes labelled in bolded letters have high risk variants associated with ASD. Genes labelled in underlined letters are syndromic and have high risk variants associated with ASD. Genes labelled in green letters are associated with syndromic ASD and this includes synaptic, intracellular signaling and transcriptional regulators

In addition to histone acetylation, another major mechanism that regulates chromatin accessibility is the methylation of histone tails (Fig. 2). Distinct histone methylation patterns in different lysine residues are known to promote either transcriptional repression or activation and have been associated with syndromic ASD [81]. However, studies that interrogate the connection between MIA, ASD pathogenesis and alterations in histone methylation patterns are limited. MIA studies in rats showed elevated levels of histone H3 tri-methylation on lysine 9 (H3K9me3) in the male offspring [82]. H3K9me3 is associated with heterochromatin formation promoting repression of gene expression [83], which suggests that MIA might lead to widespread transcriptional repression by altering the chromatin environment. However, the extent to which human fetal brains exposed to MIA could have changes in H3K9me3 levels or other histone methylation marks is largely unknown.

#### Chromatin and transcriptional regulators moonlight as immune regulatory factors.

Several chromatin and transcriptional regulators that are major genetic risk factors for ASD also have a role in the regulation of the immune system. For example, the histone methyltransferase ASH1L is a major genetic risk factor for ASD that modulates neuronal connectivity [84–87], but also regulates the polarization of Treg Cells by activating expression of SMAD3 [88]. Further, ASH1L represses IL-6 production and TLR3 triggered TNF production in macrophages in response to sepsis conditions [89], which suggests it has a major role in the inflammatory response. Like ASH1L, the transcriptional regulator FOXP1, which is a major ASD risk gene, also regulates immune Treg cells [90]. Similarly, an in-silico study of ASD-candidate genes from which interacting networks were derived showed increased representation of immune signaling pathways that included NF-kB, TNF and JNK signaling networks [91]. We further investigated the intersection between ASD risk genes and inflammation by comparing the Immunology Database and Analysis Portal (ImmPort) [92] with the Simons Foundation for Autism Research (SFARI) human gene database [93] to find overlapping immune and ASD risk genes. We identified 45 ASD risk genes that were categorized by SFARI as category 1 (high risk variants identified), category 1S (high risk variants and syndromic), and category S (syndromic ASD) that also play a role in the immune system (Fig. 3 and Supplementary Table S1). Among this group several chromatin and transcriptional regulators were identified including transcription factors FOXP1, MEF2C and SIN3; histone acetyl transferase EP300; and chromatin remodelers CHD7, CHD8, ADNP. We posit that mutations in genes encoding chromatin and transcriptional regulators that have overlapping functions in immune regulation and neurodevelopment could increase the susceptibility to inflammation in the developing fetus. Alternatively, MIA could alter the expression of genes encoding chromatin and transcriptional regulators that in turn could disrupt normal brain development, and simultaneously exacerbate the effect of inflammation on neuronal development by dysregulating immune pathways.

## HOW INFLAMMATION SHAPES THE CELLULAR LANDSCAPE LEADING TO ASD

### Brain cell types underlying the connection between ASD and inflammation

Development of the neuronal circuitry requires cross-talk between different neuronal and non-neuronal cell types. Glial cells encompassing astrocytes, oligodendrocytes, and microglia are essential for synaptic development, function, or refinement, and can directly respond to inflammatory insults. We provide an overview on how glial cells could underlie the pathogenesis of ASD.

#### Microglia

Microglia are the resident macrophage cells in the brain and have been proposed as major modulators of synaptic pruning which is essential for the refinement of neuronal circuitry [94–97]. In their resting state, microglia have a ramified morphology that is proposed to help them conduct a surveillance function in the brain [97]. In response to inflammation, microglia alter their morphology and become activated, displaying both phagocytic activity and increased cytokine production [98]. Microglia progenitors invade the developing brain during early-embryogenesis stages right after neuronal tube closure [99]. Work on rodents and non-human primates suggests that microglia control the numbers of neuronal progenitor cells (NPCs) in the developing brain [100]. Elegant genetic studies in multiple mouse models devoid of microglia or with impaired microglia function also suggest a role for embryonic microglia in the layer-specific positioning of cortical interneurons [101]. Furthermore, growing evidence suggests that microglia might also modulate neurite outgrowth. For example, genetic disruption of microglial function impaired axonal outgrowth of dopaminergic neurons in the forebrain [101]. Comparably, disruption of microglia function led to a reduction of axons in the corpus callosum [102]. This finding is relevant to the potential role of microglia dysfunction in some forms of ASD pathogenesis presenting with agenesis or hypoplasia of the corpus callosum [103–105]. Additionally, studies of postmortem brain tissue from ASD individuals demonstrated increased expression of transcription factors that regulate microglia development, which correlated with increased numbers of microglia and changes in their morphology [106, 107]. Similarly, ASD associated loss of function mutations in Neuroligin-4 (NLGN4) cause changes in microglia pruning activity, lower density of microglia, and decreased complexity of microglia morphology, which could underlie the behavioral and synaptic phenotypes of NLGN4 mutant mice [108]. In contrast, MIA animal models showed increased microglia motility [109], activation [110] and proliferation. Increased numbers of microglia or upregulation of microglial activation could potentially affect NPC proliferation [111], impair neurite outgrowth, and/or lead to over-pruning of synapses, which in turn could disrupt neuronal circuitry development [112].

#### Astrocytes and oligodendrocytes

Astrocytes and oligodendrocytes are major contributors to the proper development of neuronal circuitry and can respond to inflammatory signals. Astrocytes have a major role in synaptic modulation [113], and oligodendrocytes deposit myelin on axons which safeguards neuronal transmission [114]. In a mouse model of syndromic ASD, persistent astrocyte activation was observed [115]. More importantly, astrocyte specific mutations of known ASD risk genes have been associated with electrophysiological and sociability defects in mice [116, 117]. Similarly, studies on ASD postmortem brains showed increased astrocyte and microglial activation [118], and decreased numbers of astrocytes [119]. Alternatively, ASD pathogenesis has been associated with defects in myelination which indicates oligodendrocyte dysfunction [120]. For example, oligodendrocyte lineage specific deletion of Chd8, a major ASD genetic risk factor, alters functional connectivity and causes reduced sociability in Chd8 mutant mice [121, 122]. The role astrocytes and oligodendrocytes play in ensuring the correct establishment of brain wiring, and their capacity to respond to inflammatory events makes them prime candidates to be involved in MIA associated ASD. In fact, MIA models showed increased astrogliosis independent of the gestational day at which the immune challenge was presented [123, 124]. The essential function of astrocytes in the control of synaptogenesis [125], suggests that increased activation of astrocytes in MIA models could disrupt the normal development of the neural circuitry. Furthermore, reduced expression of oligodendroglia-related genes in MIA animal models [126, 127] correlated with white matter (nervous tissue containing myelin) abnormalities. Therefore, oligodendrocyte dysfunction could also contribute to the white matter defects observed in rodent and non-human primate MIA models [126–129], and astrocyte hyperactivation could disrupt synaptic development.

### Major inflammation pathways implicated in MIA

A large number of cytokines have been associated with ASD risk (for a detailed description please see Table 4). We focus on IL-6, IL-17A and Toll-Like Receptor (TLR) signaling as they appear to play a critical role in MIA related pathogenesis [6].

**Table 4:** Summary of immunological cytokines shown to play a role in ASD.

Cytokine	Immunological Origin	Cellular signaling effects/function	Changes linked to ASD	Citations
IL-1β	Innate immunity: secreted by monocytes & macrophages	Proinflammatory response regulation: higher expression linked to higher levels of inflammation	↑ IL-1β in serum found in individuals with ASD. ↑ levels of cerebral cortex inflammation found in ASD individuals associated with IL-1β	[21, 58, 196, 197]
IL-4	Adaptive immune response: secreted by T cells, eosinophils, basophils	Signals differentiation of Th0 cells to Th2 cells. Involved in STAT6 signaling. High expression of receptors in microglia	↑ IL-4 in peripheral blood of individuals with ASD. ↑ IL-4 in serum levels of midgestational pregnant mothers: ↑ risk of children with ASD	[51, 54]
IL-5	Adaptive immune response: secreted by Th2 cells	Stimulates differentiation and activation of B cells	↑ IL-5 in serum found in individuals with ASD	[4, 198]
IL-6	Adaptive immune response: secreted by macrophages and monocytes upon inflammatory stimulation	Stimulates differentiation of Th0 cells to Th17 cells. Activator of microglia	↑ IL-6 in serum of mothers during pregnancy: ↑ differentiation of proinflammatory IL-17 secreting Th17 cells	[10, 24]
IL-8	Adaptive immune response: secreted by macrophages, monocytes, and neutrophils	Recruiting of immune cells to inflammatory sites	Increased levels of IL-8 in ASD patients. Increases in IL-8 levels in blood also found to be associated with central nervous system damage	[59, 199]
IL-10	Adaptive + innate immune response: secreted by T cells, monocytes, and macrophages	Negative regulator of immune response and inflammation. High expression of receptors in microglia	Conflicting reports show moderately ↓IL-10 in individuals with ASD while others show ↑ levels in ASD individuals	[200, 201]
IL-17A	Adaptive immune response: secreted by Th17 cells	Proinflammatory, activates neutrophil response and recruiting, acts as inflammation signaling mediator	↑ IL-17 in serum of pregnant mothers with autistic children. Can cross placental barrier, may affect brain development through activation of microglia	[7, 24]
TNF-α	Adaptive immune response: secreted by macrophages, monocytes, t-lymphocytes, natural killer cells	Involved in regulation of immune response through inducing apoptosis and fever through pyrogenic activity. High expression of receptors in microglia	↑ TNF-α found in CSF of individuals with ASD	[202, 203]
TNF-β	Innate immune response: secreted by lymphocytes	Involved in regulation of immune response through apoptosis and affecting differentiation and proliferation	↑ TNF-β found in amniotic fluid of children later diagnosed with ASD	[53, 204]
IFN-γ	Innate and adaptive immune response: secreted by Th1 beta cells	Proinflammatory, activates macrophages and additional innate immune response. Linked to synaptic function, plasticity, and neuronal differentiation in sdeveloping brain	↑ serum levels of IFN-γ in pregnant mothers’ second trimester: associated with ↑ risk of ASD. IFN-γ treatment leads to ↓ synaptic density and neurite outgrowth in stem cell derived neurons	[164, 167, 168, 205]

Dysregulation of these cytokines have been shown to play a role in development and pathogenesis of ASD

#### IL-6 in neurodevelopment and ASD risk

A study of maternal-fetal transfer at the placenta identified IL-6 as one of the cytokines that could be transferred to the fetal circulation [130]. Therefore, based on the immaturity of the developing fetal blood–brain barrier [131], it is possible that IL-6 can exert its effect directly in the developing brain. The importance of IL-6 to mediate MIA impact on neuronal development is further highlighted by the fact that after in utero exposure to MIA, IL-6 knockout mice lacked ASD-like behavioral phenotypes compared to their wildtype littermates [10]. Injection of IL-6 on day 12.5 pregnant mice lead to electrophysiological deficits in pre-pulse inhibition in the adult offspring [10], as well as the disruption of the canonical balance of excitatory/inhibitory synaptic transmissions [132]. In normal immunity, IL-6 is released in response to an infection, leading to the differentiation of Th0 cells to activated Th17 cells that then release IL-17A [24], which has been proposed to be a direct effector of MIA phenotypes [7, 133].

#### IL-17A in neurodevelopment and ASD risk

Astrocytes and microglia express the IL-17A receptor (IL-17RA) and could respond directly to elevated levels of IL-17A [134]. IL-17A might also exert its effect directly on neurons, as upregulated expression of IL-17RA in mouse cortical neurons has been observed in response to MIA [7]. In the absence of maternal inflammation, in-utero injection of recombinant IL-17A into the cortices of embryonic day 14.5 (E14.5) mice was shown to recapitulate brain anatomical and behavioral phenotypes associated with MIA [7]. Moreover, inactivation of the IL-17RA either by antibody injection or using conditional genetics was sufficient to rescue MIA induced ASD-like behaviors [7, 133]. Therefore, the IL-17A pathway might play a crucial role as an effector of MIA in the developing brain.

#### TLRs as mediators of immune response in the developing brain

The effect of inutero inflammation during pregnancy has been modeled in rodents and non-human primates using different strategies to elicit an immune response. Poly (I:C) and LPS are commonly used immunogens that target different TLRs; therefore, an emerging cellular mechanism that could be underlying some of the effects of inflammation in the developing brain is the activation of the TLR signaling pathway [135]. Poly (I:C), a molecule that resembles double stranded RNA to mimic viral infection, has been found to interact through TLR3, while LPS or lipopolysaccharide (immunostimulant structures that exist in some bacterial outer membranes) mimics bacterial infections by activating TLR4 [136]. Activation of the TLR signaling pathway leads to the transcriptional activation and subsequent production of pro-inflammatory cytokines and interferons. In utero exposure to poly (I:C) during mid-gestational period induced an acute inflammatory response resulting in increased expression of cytokines in the fetal brain [137], which correlates with overproduction of earlier born cortical excitatory projection neurons [138]. Furthermore, optogenetic studies of MIA offspring exposed to poly (I:C) in utero showed increased synaptic strength in glutamatergic projections between the medial prefrontal cortex and the basal amygdala, which is a circuit implicated in ASD [139]. Alterations in the numbers of cortical excitatory neurons could disrupt the excitation/inhibition balance in the brain which has been proposed as a mechanism of disease in ASD [140]. Comparatively, in a rat model of MIA, LPS stimulation lead to downregulation of genes that regulate migration of GABAergic interneurons [141], which could disrupt the brain architecture and impair the normal development of neuronal circuitry. Changes in distribution or numbers of different neuronal subtypes could be a common mechanism of disease associated with MIA models across different species [138, 141]. LPS also activates astrocytes and microglia, by inducing the release of IL-6 which in turn leads to increase production of IL-1β [142]. Based on the role astrocytes and microglia play in synaptic function and synapse remodeling, their activation could result in microcircuit defects.

## IPSC-BASED SYSTEMS AS A MODELING PLATFORM FOR THE CONTRIBUTION OF INFLAMMATION TO ASD ETIOLOGY

**Figure 4:**
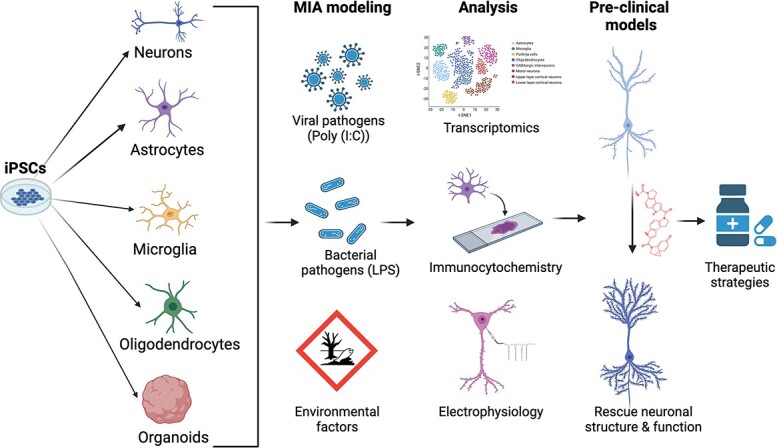
**Modeling MIA in human systems.** Stem cell technology allows the capacity to test the effects of inflammatory agents on different brain cell types or brain organoids. Using a combination of multi-disciplinary approaches will allow for the characterization of the cellular, molecular and physiological phenotypes in neural cells or organoids after inflammation. The phenotypes identified could be used to test drugs that are able to rescue the phenotypes which could then lead to the development of therapeutic strategies to treat neurological disorders associated with MIA

In humans it is not possible to model how inflammation affects the early development of neuronal connectivity in vivo. Human iPSC-derived two dimensional (2D) and three dimensional (3D) neural models constitute an unprecedented opportunity to tease apart the mechanisms that might be impaired by inflammation during in utero human brain development (Fig. 4). Furthermore, iPSC-based models provide us with a window into otherwise inaccessible tissue, allowing us to test the susceptibility to inflammation associated with ASD pathogenic mutations, as patient derived iPSCs retain the genetic background of each individual [143]. Transcriptional studies of 2D and 3D iPSC-derived neural models resemble in vivo developmental stages equivalent to mid to late fetal brain development [144–146].

### Two-dimensional iPSC-based experimental systems untangling the complexity of the brain

Robust protocols have been established to differentiate stem cells into multiple neuronal and non-neuronal lineages including: NPCs [147], cortical excitatory neurons [147], inhibitory interneurons [148–150], microglia [151–154], astrocytes [155–158], and oligodendrocytes [159–162]. Therefore, the capacity to generate different neural and glial cell types makes it possible to assess cell-autonomous and non-cell autonomous responses to inflammatory challenges. For example, human iPSC-derived microglia co-cultured with iPSC-derived cortical neurons released multiple cytokines in response to an inflammatory challenge [163]. In an iPSC-model of Parkinson’s disease that used a co-culture of microglia and dopaminergic neurons, microglia activated by IFN-γ caused neurite outgrowth defects [164]. In addition to microglial models, generation of astrocytes that elicit a pro-inflammatory response to IL-1β and TNF-α could serve as an additional model to interrogate the effects of inflammation on developing neurons in vitro [165]. Interestingly, iPSC-derived astrocytes from idiopathic ASD individuals produced higher levels of IL-6 compared to control astrocytes [166]. Further, co-culturing of iPSC-derived astrocytes from ASD individuals with control neurons impaired neuronal development, which was rescued by inhibition of IL-6 [166]. Therefore, elevated IL-6 production in astrocytes suggests that immune activation could be a downstream phenomenon that is non-cell autonomous and could exacerbate the neural phenotypes associated with ASD pathogenesis. However, the iPSCs used in this study were obtained from donors with no known genetic lesions, which does not preclude the possibility that the altered IL-6 levels might result from specific mutations impacting this pathway.

While inflammation could primarily exert its effects in the nervous system primarily through the microglia/astrocyte axis, the direct effect of inflammatory pathways in neurons or NPCs cannot be discounted. For instance, human dopaminergic-like neurons derived from mesencephalic stem cells were treated with five different cytokines elevated in mid-gestational serum samples of mothers from children with ASD, and showed reduced neurite outgrowth and diminished synaptic density in response to a mixture of TNF-α, IL-1β, IL-6, IL-17A and IFN-γ [167]. The cellular phenotypes appeared to be mediated by activation of the NFκ-B signaling pathway [167]. Similarly, IFN-γ treated iPSC-derived NPCs lead to excitatory neurons with impaired neurite outgrowth that correlated with differential expression of known ASD risk genes, as well as activation of IFN-γ response genes like JAK/STAT [168]. Follow up studies showed that glutamatergic immature neurons acutely exposed to IFN-γ had activation of STAT1-signaling, increased levels of the major histocompatibility complex I (MHCI), which is involved in synaptic pruning in mice [169], and reduced the levels of SYNAPSIN proteins in the cell soma [170]. This suggests that IFN-γ exposure could dysregulate human neuronal structure and function of glutamatergic neurons [167]. In contrast, human iPSC-derived NPCs exposed to IL-17A showed increased neuronal differentiation in response to retinoic acid treatment, but no measurements of neurite outgrowth were reported [171]. Human NPCs exposed to IL-17A showed activation of mTOR and ERK1/2 downstream signaling but surprisingly NFκ-B signaling did not appear to be activated [171].

Finally, accumulation of perivascular macrophages and monocytes in response to inflammation [15] could also contribute to the neural phenotypes observed. Therefore, future studies that incorporate multiple cellular lineages should consider incorporating these cell types [63]. The development of protocols to differentiate macrophages from iPSCs increases the feasibility of evaluating not only the contribution of neural and glial lineages to ASD pathogenesis but also of macrophages to the human phenotypes [172–174] . In summary, development of 2D neural models provide a platform to study the effect of inflammatory signals using a reductionist approach. Despite their relevance to mechanistic studies, 2D models are unable to recapitulate the cellular complexity and the spatiotemporal dynamics of the developing brain, which suggests the relevance of incorporating 3D systems into further studies.

### Three-dimensional iPSC-based systems provide cellular complexity to model brain development

The development of 3D-human brain organoids that can recapitulate in part the temporal and spatial organization of the developing brain constitutes a major advancement in how we study human brain development [175, 176]. Generation of brain region specific organoids allows us to ask questions relevant to early development specific to each brain region [177, 178]. Moreover, brain region specific organoids can be used to generate ‘assembloids’ to interrogate how different brain regions interact with one another during development of neuronal circuits [179, 180]. Most of the brain or neural organoid models resemble primarily the transcriptional profile of mid-fetal gestational stages of brain development, which makes them an ideal model to address how inflammation affects brain development at these stages [145, 181]. Recent work suggests that long term maturation of forebrain cortical organoids grown longer than 300 days shows increased overlap in terms of transcriptomic profiles with postnatal stages of brain development [182]. Therefore, using a long-term maturation protocol, cortical organoids could be treated at early stages with pro-inflammatory cytokines, and analyzed later to assess the long-term effects of inflammation on the transcriptomic profiles of late-stage organoids.

Several lines of evidence highlight the relevance of 3D neural systems to model MIA in human systems. First, cerebral organoids express receptors to cytokines such as IL-17A, which are relevant to MIA studies [183]. Second, transcriptomic studies of human dorsal forebrain patterned organoids showed that 6-month-old organoids were comparable at the transcriptional level to fetal mid-gestation stages at post conception weeks (pcw) 13 to 15 and had a robust immune transcriptomic signature with differential expression of INF-γ□signaling pathway genes [181]. The human fetal brain expresses the canonical IL-6 receptors in microglia. However, GP130, which is a non-canonical receptor for IL-6, is highly expressed in radial glial cells, a major NPC subtype [184]. This suggests a cell-type specific function for IL-6 downstream signaling in radial glia. In fact, forebrain organoids were treated with a chimeric protein resembling IL-6 bound to its receptor that activated GP130 and led to defects in cortical lamination of the organoids [184]. Therefore, these studies using 3D based systems highlight the importance of assessing the effects of inflammation on multiple brain regions and cell types and provide a better understanding of how maternal infections during pregnancy could affect in utero and early postnatal brain development. However, studies using brain organoids do not entirely recapitulate the more complex interactions that occur in the brain microenvironment in vivo, which could be addressed using xenotransplantation models.

### iPSC-based human and mouse brain chimeric systems

The brain microenvironment could contribute to cell type specific programs. For example, microglia grown in vitro have a distinct transcriptomic profile compared to in vivo microglia [185]. However, 2D systems are insufficient to recapitulate complex cell–cell signaling events. Similarly, while 3D systems have higher cellular complexity in vitro, they are limited in terms of recreating the contribution of the larger brain microenvironment to cellular phenotypes or integration into proper neuronal circuitry. The use of chimeric models in which human iPSC-derived NPCs, microglia progenitor cells, brain organoids, or a combination of microglia and brain organoids are transplanted into the brain of immunosuppressed mice provide an advantage to in vitro models and could address how the surrounding microenvironment contributes to cellular development and function [185–187]. However, this is also limited as the cross talk between multiple cell types from different species can be distinct from the human brain.

Transplantation of microglia progenitors has been done at early postnatal stages achieving various levels of chimerism and have been shown to functionally integrate into the chimeric mouse brain [187, 188]. For example, following chemically induced depletion of endogenous murine microglia in newborn animals, human iPSC-derived microglial progenitors were transplanted to mouse brains at postnatal day 4, showing close to 80% chimerism between 3 to 6 months of age [188]. However, for the microglia to thrive, the immunodeficient mouse models need to be humanized by expressing specific human molecules such as human CSF1 [185, 187]. Therefore, there is a need to recreate a human brain-like microenvironment in vivo. Human iPSC-derived cortical organoids were colonized in vitro by human iPSC-derived microglia and transplanted into an adult mouse brain allowing for the infiltration of the host vasculature into the transplanted human organoids [186]. This human microglia-organoid chimeric mouse brain model led to the development of transcriptomic signatures that closely resemble the signatures identified in human brain tissue at the single cell level, and demonstrated that the human microglia are capable of surveilling the human brain-like environment in response to injury [186]. Importantly, these methods may be used for patient-derived iPSC applications, such as observing how genetic mutations influence or are influenced by the surrounding brain microenvironment.

### Limitations of iPSC-based systems to study the contribution of inflammation to ASD etiology

Despite the advantages that iPSC-based systems bring to the study of the intersection of the neuroimmune mechanisms and autism etiology, there are limitations to these systems in addressing the elaborate contribution of the immune response to human neuronal development. For instance, while one can study in isolation the contribution of different pro-inflammatory cytokines to different neural cell types, these systems fall short in terms of the complexity of the immune response or the role of the placenta in inflammation. Additionally, despite the larger cellular heterogeneity of the 3D systems, they lack vascularization which is an integral part of proper brain development. Finally, while xenotransplantation technology of human organoids into the chimeric mouse brain provides a human brain-like environment, a concern is the extent to which the host tissue can itself exert an effect on the transplanted human tissue and alter human-specific phenotypes.

## FINAL REMARKS

Defining alterations during development of the precise mechanisms associated with MIA and ASD pathogenesis will require the integration of multiple experimental systems using in vivo animal models and in vitro human cellular models. However, for in vitro systems to truly mirror the in vivo MIA phenotypes, the influence of the genetic background at the cellular, molecular, and physiological levels will need to be considered. Thus, using iPSCs derived from multiple unrelated individuals will account for the inherent variability in iPSCs due to each individual unique genetic background and retention of donor-specific epigenetics [189]. Similarly, studies that incorporate male and female iPSC-based models will be important to gain mechanistic insight into sex-specific differences in ASD. Furthermore, patient-derived iPSCs with pathogenic mutations associated with ASD that are then subjected to inflammatory conditions could inform whether exposure to inflammatory signals exacerbate ASD-related cellular and molecular phenotypes [190].

Besides the mechanistic insight that iPSC-based studies offer, these systems can also provide a platform to conduct high-throughput drug screening for the rapid identification of rationally designed therapeutics [191, 192]. For example, neurite outgrowth defects are associated with different ASD subtypes [193, 194], and were used as a readout metric in a high-throughput screen to analyze the effects of over 4000 small molecules on neurite outgrowth [195]. This approach identified 50 compounds that modulated neurite outgrowth without cytotoxic effects [195]. Since 2D neurons exposed to a cytokine cocktail showed reduced neurite outgrowth [167], future high-throughput screening for compounds that restore neurite length could identify rescue strategies in MIA iPSC-based experimental systems. Additionally, as the field moves forward it will be important to consider that there is also non-genetic variation associated with iPSC derived models including differences in passage number, culturing, and differentiation protocols, therefore, there is a need for a concerted effort across research groups pursuing these types of studies to use standardized methodologies.

In this review we have provided a broad overview of the cellular and molecular mechanisms that could underlie the role of inflammation in ASD pathogenesis. A critical question that remains in the field is whether alterations in the immune system are associated phenomena that nonetheless contribute to the overall pathobiology of ASD. Future studies that integrate patient derived iPSC-based models with the study of genomic/epigenomic signatures at a single cell resolution will allow us to further dissect the contribution of genetic and environmental risk factors like inflammation to ASD risk. Finally, the development of human cellular models in combination with in-depth mechanistic studies will be important to establish robust pre-clinical models to test mechanistic-based therapeutic strategies (Fig. 4).

## Supplementary Material

Web_Material_kvae003
