# Supplementary material for: Neuroimmune mechanisms in autism etiology - untangling a complex problem using human cellular models
Source: Oxf Open Neurosci. 2024 Feb 22;3:kvae003. doi: 10.1093/oons/kvae003 (PMC11044813; doi:10.1093/oons/kvae003)
Supplement: Web_Material_kvae003 [file web_material_kvae003.pdf]

Supplementary Table S1

SFARI autism risk genes identified in the immune database

ID	Species	Ensembl - ID	Entrez ID	Gene name	gene full name	gene name synonyms	Chromosome Strand	Chromosome	Chromosome band	GO-Terms	Cellular Localization
81086	Homo sapiens	ENSG00000101126	23394	ADNP	activity-dependent neuroprotector homeobox	ADNP1|MRD28	-1	20	q13.13	GO:0003677|DNA binding|molecular_function	Nucleus
32178	Homo sapiens	ENSG00000184254	220	ALDH1A3	aldehyde dehydrogenase 1 family, member A3	ALDH1A6|ALDH6|MCOP8|RALDH3	1	15	q26.3	GO:0002072|optic cup morphogenesis involv	Cytoplasm
47029	Homo sapiens	ENSG00000182287	8905	AP1S2	adaptor-related protein complex 1, sigma 2 subuni	MGC:1902|MRX59|MRXS21|MRXS5|MRXSF|PGS|SIGMA1B	-1	X	p22.2	GO:0000139|Golgi membrane|cellular_compo	Plasma membrane
73041	Homo sapiens	ENSG00000131089	23229	ARHGEF9	Cdc42 guanine nucleotide exchange factor (GEF)	COLLYBISTIN|EIEE8|HPEM-2|PEM-2|PEM2	-1	X	q11.1	GO:0005089|Rho guanyl-nucleotide exchang	Cytoplasm
52891	Homo sapiens	ENSG00000119866	53335	BCL11A	B-cell CLL/lymphoma 11A (zinc finger protein)	BCL11A|IDBG-52891|ENSG00000119866|53335	-1	2	p16.1	GO:0000122|negative regulation of transcrip	Nucleus
44351	Homo sapiens	ENSG00000157764	673	BRAF	v-raf murine sarcoma viral oncogene homolog B1	B-RAF1|BRAF1|NS7|RAFB1	-1	7	q34	GO:0000165|MAPK cascade|biological_proc	Nucleus
22967	Homo sapiens	ENSG00000171316	55,636	CHD7	chromodomain helicase DNA binding protein 7	CHD7|IDBG-22967|ENSG00000171316|102724260,55636	1	8	q12.2	GO:0001501|skeletal system development|b	Nucleus
2645	Homo sapiens	ENSG00000100888	57680	CHD8	chromodomain helicase DNA binding protein 8	CHD8|IDBG-2645|ENSG00000100888|57680	-1	14	q11.2	GO:0001701|in utero embryonic developmen	Nucleus
46789	Homo sapiens	ENSG00000174469	26047	CNTNAP2	contactin associated protein-like 2	AUTS15|CASPR2|CDFE|NRXN4|PTHSL1	1	7	q35	GO:0005515|protein binding|molecular_funct	Cell surface
58519	Homo sapiens	ENSG00000066032	1496	CTNNA2	catenin (cadherin-associated protein), alpha 2	CAP-R|CAPR|CT114|CTNR	1	2	p12	GO:0005198|structural molecule activity|mol	Cell surface
99280	Homo sapiens	ENSG00000198719	28514	DLL1	delta-like 1 (Drosophila)	DLL1|IDBG-99280|ENSG00000198719|28514	-1	6	q27	GO:0001709|cell fate determination|biologica	Extracellular
93214	Homo sapiens	ENSG00000108001	253738	EBF3	early B-cell factor 3	EBF3|IDBG-93214|ENSG00000108001|253738	-1	10	q26.3	GO:0003677|DNA binding|molecular_function	Nucleus
8992	Homo sapiens	ENSG00000100393	2033	EP300	E1A binding protein p300	KAT3B|p300|RSTS2	1	22	q13.2	GO:0000086|G2/M transition of mitotic cell c	Nucleus
44357	Homo sapiens	ENSG00000114861	27086	FOXP1	forkhead box P1	FOXP1|IDBG-44357|ENSG00000114861|27086	-1	3	p13	GO:0000122|negative regulation of transcrip	Nucleus
107346	Homo sapiens	ENSG00000143641	2590	GALNT2	UDP-N-acetyl-alpha-D-galactosamine:polypeptide N	GalNAc-T2	1	1	q42.13	GO:0000139|Golgi membrane|cellular_compo	Plasma membrane
90667	Homo sapiens	ENSG00000172534	3054	HCFC1	host cell factor C1 (VP16-accessory protein)	CFF|HCF-1|HCF1|HFC1|MRX3|PPP1R89|VCAF	-1	X	q28	GO:0000122|negative regulation of transcrip	Nucleus
84594	Homo sapiens	ENSG00000068024	9759	HDAC4	histone deacetylase 4	AHO3|BDMR|HA6116|HD4|HDAC-4|HDAC-A|HDACA	-1	2	q37.3	GO:0000118|histone deacetylase complex|c	Nucleus
75353	Homo sapiens	ENSG00000165478	220,296	HEPACAM	hepatic and glial cell adhesion molecule	HEPACAM|IDBG-75353|ENSG00000165478|220296,641654	-1	11	q24.2	GO:0005515|protein binding|molecular_funct	Cell surface
19708	Homo sapiens	ENSG00000083168	7994	KAT6A	K(lysine) acetyltransferase 6A	MOZ|MYST3|RUNXBP2|ZC2HC6A|ZNF220	-1	8	p11.21	GO:0000786|nucleosome|cellular_componen	Nucleus
33077	Homo sapiens	ENSG00000081189	4208	MEF2C	myocyte enhancer factor 2C	C5DELq14.3|DEL5q14.3	-1	5	q14.3	GO:0000122|negative regulation of transcrip	Nucleus
89258	Homo sapiens	ENSG00000198793	2475	MTOR	mechanistic target of rapamycin (serine/threonine	FRAP|FRAP1|FRAP2|RAFT1|RAPT1	-1	1	p36.22	GO:0000139|Golgi membrane|cellular_compo	Nucleus
31849	Homo sapiens	ENSG00000160877	112939	NACC1	nucleus accumbens associated 1, BEN and BTB (	NACC1|IDBG-31849|ENSG00000160877|112939	1	19	p13.13	GO:0005515|protein binding|molecular_funct	Nucleus
39462	Homo sapiens	ENSG00000196712	4,763	NF1	neurofibromin 1	NFNS|VRNF|WSS	1	17	q11.2	GO:0000165|MAPK cascade|biological_proc	Nucleus
16758	Homo sapiens	ENSG00000164190	25836	NIPBL	Nipped-B homolog (Drosophila)	CDLS|CDLS1|IDN3|IDN3-B|Scc2	1	5	p13.2	GO:0000122|negative regulation of transcrip	Nucleus
23394	Homo sapiens	ENSG00000179364	23241	PACS2	phosphofurin acidic cluster sorting protein 2	PACS-2|PACS1L	1	14	q32.33	GO:0005739|mitochondrion|cellular_compone	Unknown
65610	Homo sapiens	ENSG00000149269	5058	PAK1	p21 protein (Cdc42/Rac)-activated kinase 1	PAKalpha	-1	11	q14.1	GO:0001666|response to hypoxia|biological_	Nucleus
79092	Homo sapiens	ENSG00000165194	57526	PCDH19	protocadherin 19	PCDH19|IDBG-79092|ENSG00000165194|57526	-1	X	q22.3	GO:0005509|calcium ion binding|molecular_f	Plasma membrane
93358	Homo sapiens	ENSG00000146247	55023	PHIP	pleckstrin homology domain interacting protein	PHIP|IDBG-93358|ENSG00000146247|55023	-1	6	q14.1	GO:0001932|regulation of protein phosphory	Nucleus
79933	Homo sapiens	ENSG00000148606	11128	POLR3A	polymerase (RNA) III (DNA directed) polypeptide A	ADDH|HLD7|hRPC155|RPC1|RPC155	-1	10	q22.3	GO:0001056|RNA polymerase III activity|mo	Nucleus
64389	Homo sapiens	ENSG00000198914	5455	POU3F3	POU class 3 homeobox 3	BRN1|OTF8	1	2	q12.1	GO:0001822|kidney development|biological_	Nucleus
44693	Homo sapiens	ENSG00000113575	5515	PPP2CA	protein phosphatase 2, catalytic subunit, alpha isc	PP2Ac|PP2CA|PP2Calpha|RP-C	-1	5	q31.1	GO:0000159|protein phosphatase type 2A c	Nucleus
4215	Homo sapiens	ENSG00000184304	5587	PRKD1	protein kinase D1	PKC-MU|PKCM|PKD|PRKCM	-1	14	q12	GO:0001525|angiogenesis|biological_proces	Nucleus
1197	Homo sapiens	ENSG00000100033	5625	PRODH	proline dehydrogenase (oxidase) 1	HSPOX2|PIG6|POX|PRODH1|PRODH2|TP53I6	-1	22	q11.21	GO:0004657|proline dehydrogenase activity	Cytoplasm
65421	Homo sapiens	ENSG00000197170	5718	PSMD12	proteasome (prosome, macropain) 26S subunit, no	p55|Rpn5	-1	17	q24.2	GO:0000082|G1/S transition of mitotic cell c	Nucleus
81409	Homo sapiens	ENSG00000171862	5728	PTEN	phosphatase and tensin homolog	10q23del|BZS|CWS1|DEC|GLM2|MHAM|MMAC1|PTEN1|TEP1	1	10	q23.31	GO:0000079|regulation of cyclin-dependent	Nucleus
8115	Homo sapiens	ENSG00000136238	5879	RAC1	ras-related C3 botulinum toxin substrate 1 (rho fan	p21-Rac1|Rac-1|TC-25	1	7	p22.1	GO:0000139|Golgi membrane|cellular_compo	Plasma membrane
33417	Homo sapiens	ENSG00000164754	5885	RAD21	RAD21 homolog (S. pombe)	CDLS4|hHR21|HR21|HRAD21|MCD1|NXP1|SCC1	-1	8	q24.11	GO:0000228|nuclear chromosome|cellular_c	Nucleus
13210	Homo sapiens	ENSG00000006451	5898	RALA	v-ral simian leukemia viral oncogene homolog A (ra	RAL	1	7	p14.1	GO:0000910|cytokinesis|biological_process#	Cell surface
31870	Homo sapiens	ENSG00000176406	9699	RIMS2	regulating synaptic membrane exocytosis 2	OBOE|RAB3IP3|RIM2	1	8	q22.3	GO:0005515|protein binding|molecular_funct	Cell surface
39370	Homo sapiens	ENSG00000181481	84282	RNF135	ring finger protein 135	RNF135|IDBG-39370|ENSG00000181481|84282	1	17	q11.2	GO:0004842|ubiquitin-protein transferase ac	Cytoplasm
14920	Homo sapiens	ENSG00000069667	6095	RORA	RAR-related orphan receptor A	NR1F1|ROR1|ROR2|ROR3|RZR-ALPHA|RZRA	-1	15	q22.2	GO:0001222|transcription corepressor bindir	Nucleus
50812	Homo sapiens	ENSG00000177189	6,197	RPS6KA3	ribosomal protein S6 kinase, 90kDa, polypeptide 3	CLS|HU-3|ISPK-1|MAPKAPK1B|MRX19|p90-RSK2|pp90RSK2	-1	X	p22.12	GO:0000287|magnesium ion binding|molecula	Nucleus
4715	Homo sapiens	ENSG00000142178	150094	SIK1	salt-inducible kinase 1	SIK1|IDBG-4715|ENSG00000142178|150094	-1	21	q22.3	GO:0000287|magnesium ion binding|molecula	Nucleus
23397	Homo sapiens	ENSG00000169375	25942	SIN3A	SIN3 transcription regulator homolog A (yeast)	SIN3A|IDBG-23397|ENSG00000169375|25942	-1	15	q24.2	GO:0000122|negative regulation of transcrip	Nucleus
39801	Homo sapiens	ENSG00000110436	6506	SLC1A2	solute carrier family 1 (glial high affinity glutamate	EAAT2|GLT-1	-1	11	p13	GO:0005313|L-glutamate transmembrane tra	Plasma membrane
86143	Homo sapiens	ENSG00000136854	6812	STXBP1	syntaxin binding protein 1	MUNC18-1|NSEC1|P67|RBSEC1|UNC18	1	9	q34.11	GO:0000149|SNARE binding|molecular_funct	Plasma membrane
49321	Homo sapiens	ENSG00000067715	6857	SYT1	synaptotagmin I	P65|SVP65|SYT	1	12	q21.2	GO:0000149|SNARE binding|molecular_funct	Cell surface
1419	Homo sapiens	ENSG00000184058	6899	TBX1	T-box 1	CAFS|CATCH22|CTHM|DGCR|DGS|DORV|TBX1C|TGA|VCF|VC	1	22	q11.21	GO:0001525|angiogenesis|biological_proces	Nucleus
10062	Homo sapiens	ENSG00000131653	84231	TRAF7	TNF receptor-associated factor 7, E3 ubiquitin prot	TRAF7|IDBG-10062|ENSG00000131653|84231	1	16	p13.3	GO:0000151|ubiquitin ligase complex|cellular	Vesicles and Plasma m
82749	Homo sapiens	ENSG00000153827	9320	TRIP12	thyroid hormone receptor interactor 12	TRIP-12|ULF	-1	2	q36.3	GO:0004842|ubiquitin-protein transferase ac	Nucleus
9819	Homo sapiens	ENSG00000103197	7249	TSC2	tuberous sclerosis 2	LAM|PPP1R160|TSC4	1	16	p13.3	GO:0001666|response to hypoxia|biological_	Nucleus
4274	Homo sapiens	ENSG00000114062	7337	UBE3A	ubiquitin protein ligase E3A	ANCR|AS|E6-AP|EPVE6AP|HPVE6A	-1	15	q11.2	GO:0000502|proteasome complex|cellular_c	Nucleus
36944	Homo sapiens	ENSG00000130477	23025	UNC13A	unc-13 homolog A (C. elegans)	Munc13-1	-1	19	p13.11	GO:0001956|positive regulation of neurotran	Cell surface
57113	Homo sapiens	ENSG00000124486	8239	USP9X	ubiquitin specific peptidase 9, X-linked	DFFRX|FAF|FAM|MRX99	1	X	p11.4	GO:0000122|negative regulation of transcrip	Plasma membrane
19992	Homo sapiens	ENSG00000154767	7508	XPC	xeroderma pigmentosum, complementation group C	p125|RAD4|XP3|XPCC	-1	3	p25.1	GO:0000404|heteroduplex DNA loop binding	Nucleus
23145	Homo sapiens	ENSG00000170027	7532	YWHAG	tyrosine 3-monooxygenase/tryptophan 5-monooxy	14-3-3GAMMA|PPP1R170	-1	7	q11.23	GO:0000086|G2/M transition of mitotic cell c	Plasma membrane
19992	Homo sapiens	ENSG00000154767	7508	XPC	xeroderma pigmentosum, complementation group C	p125|RAD4|XP3|XPCC	-1	3	p25.1	GO:0000404|heteroduplex DNA loop binding	Nucleus
23145	Homo sapiens	ENSG00000170027	7532	YWHAG	tyrosine 3-monooxygenase/tryptophan 5-monooxy	14-3-3GAMMA|PPP1R170	-1	7	q11.23	GO:0000086|G2/M transition of mitotic cell c	Plasma membrane
